# Abstract of Meteorological Observations for March, 1856, Made at Philadelphia, Pa.

**Published:** 1856-05

**Authors:** James A. Kirkpatrick


*Abstract of Meteorological Observations for March, 1856, made at Philadelphia, Pa. Latitude 39° 57′ 28″ N., Longitude 75° 10′ 40″ W. from Greenwich.* By PROF. JAMES A. KIRKPATRICK.

| 1856. March. | Barometer. Daily Mean | Barometer. Mean Daily Range. | Thermom. Daily Mean | Thermom. Mean Daily Range | Rel. Humid. 2 P.M. | Force of Vapor 2 P.M. | Dew Point 2 P.M. | Rain & Melted Snow. | Prevailing Winds. | Remarks. |
|---|---|---|---|---|---|---|---|---|---|---|
| | Inches. | Inches. | Deg. | Deg. | Hunds. | Inches. | Deg. | Inches. | Points. | |
| 1 | 29.886 | .222 | 32.8 | 2.2 | 69 | .142 | 26.2 | 1.200 | (Var.) | Morning *fog ;* afternoon and evening *snow ;* night *rain.* |
| 2 | 29.354 | .532 | 37.3 | 4.5 | 52 | .150 | 27.5 | | W. | Morning *rain*; afternoon cloudy; evening clear. *Barometer lowest* 29.234. |
| 3 | 29.922 | .569 | 29.0 | 8.3 | 70 | .132 | 24.6 | | W. | Morning and evening clear; afternoon cloudy. |
| 4 | 29.877 | .255 | 37.7 | 10.7 | 69 | .214 | 36.2 | | SW. | Clear; evening *rain* for about five minutes. |
| 5 | 29.863 | .124 | 29.3 | 9.7 | 60 | .113 | 21.0 | | W. | Clear. |
| 6 | 29.599 | .264 | 33.2 | 6.2 | 59 | .163 | 29.5 | | SW. | Morning and afternoon cloudy ; 5¼, P. M., a few flakes of *snow ;* ev'n. clear. |
| 7 | 29.847 | .248 | 27.3 | 8.2 | 51 | .095 | 17.2 | | W. | Clear. |
| 8 | 29.722 | .175 | 32.8 | 8.2 | 37 | .098 | 17.9 | | (Var.) | Clear. |
| 9 | 29.918 | .196 | 15.2 | 18.0 | 52 | .052 | 4.0 | 0.037 | NW. | Cloudy; evening and night *snow.* |
| 10 | 29.931 | .041 | 11.5 | 4.0 | 47 | .041 | -1.2 | | W. | Morning and evening clear; afternoon cloudy. *Thermometer lowest* 4°. |
| 11 | 29.692 | .239 | 26.8 | 15.3 | 62 | .127 | 23.7 | | (Var.) | Cloudy. |
| 12 | 29.642 | .136 | 29.3 | 3.2 | 61 | .120 | 22.4 | | WSW. | Clear. |
| 13 | 29.837 | .195 | 31.5 | 2.2 | 63 | .144 | 26.6 | | W. | Morning clear; afternoon and evening cloudy. |
| 14 | 29.901 | .064 | 33.2 | 1.7 | 46 | .110 | 20.4 | | NW. | Clear. |
| 15 | 29.916 | .017 | 37.0 | 4.2 | 59 | .163 | 29.5 | | W. | Morning a light fall of *snow ;* evening clear. |
| 16 | 29.915 | .028 | 33.2 | 3.8 | 95 | .201 | 34.6 | 0.013 | W. | Morning and afternoon *snow*; evening clear. |
| 17 | 30.022 | .107 | 35.0 | 4.2 | 42 | .112 | 20.8 | | W. | Clear. |
| 18 | 30.045 | .043 | 39.3 | 4.3 | 42 | .142 | 26.2 | | NW. | Cloudy. |
| 19 | 29.720 | .325 | 35.5 | 5.2 | 81 | .194 | 3[illegible].7 | 0 491 | (Var.) | Morning *snow* about 6 inches deep, drizzling occasionally; evening clear. |
| 20 | 29.634 | .086 | 41.2 | 6.7 | 71 | .246 | 39.8 | | SW. | Cloudy; a few drops of *rain* at 6, P. M. *Thermometer highest* 49°. |
| 21 | 29.600 | .045 | 42.7 | 2.8 | 63 | .212 | 36.0 | | (Var.) | Cloudy. *Delaware River open, ice floating in large cakes.* |
| 22 | 29.643 | .043 | 40.7 | 2.0 | 50 | .157 | 28.6 | | NE. | Cloudy. |
| 23 | 29.710 | .067 | 41.3 | 1.3 | 41 | ·133 | 24.7 | | (Var.) | Morning and afternoon clear; evening cloudy. [3½ to 8, P.M., *rain.*] |
| 24 | 29.538 | .173 | 40.0 | 4.7 | 86 | .214 | 36.2 | 0·293 | SW. | 11½, A. M., *rain ;* 2, P. M., *hail,* white, and averaging ¼ inch in diameter; |
| 25 | 29.658 | .179 | 40.2 | 2.5 | 59 | .163 | 29.5 | | NW. | Cloudy. |
| 26 | 29.673 | .067 | 39.5 | 2.0 | 46 | .137 | 25.4 | | W. | Clear. |
| 27 | 29.541 | .132 | 35.7 | 3.8 | 46 | .110 | 20.4 | | NW. | orning and evening clear; afternoon cloudy. |
| 28 | 29.619 | .080 | 29.3 | 6.3 | 49 | .089 | 15.8 | 0.005 | NW. | Morning *snow :* afternoon and evening cloudy. |
| 29 | 29.751 | .130 | 34.0 | 4.7 | 38 | .093 | 16.9 | | W. | Clear. |
| 30 | 29.893 | .142 | 32.0 | 2.0 | 45 | .103 | 19.0 | | W. | Clear. |
| 31 | 30.080 | .187 | 30.5 | 1.8 | 61 | .129 | 24.0 | | NW. | Morning and afternoon cloudy; evening clear. *Barometer highest* 30.161. |
| Means for Mar., 1856 | 29.773 | .165 | 33.4 | 5.3 | 57 | .139 | 24.4 | 2·039 | N. 80° 47′ W. 54-100 | |
| Means for Mar., 5 yrs. | 29.827 | .169 | 39.9 | 5.5 | 54 | .163 | 31.0 | 2.475 | N. 69° 11′ W. 35-100. | |

